# Melatonin, Its Beneficial Effects on Embryogenesis from Mitigating Oxidative Stress to Regulating Gene Expression

**DOI:** 10.3390/ijms22115885

**Published:** 2021-05-30

**Authors:** Dmitry Ivanov, Gianluigi Mazzoccoli, George Anderson, Natalia Linkova, Anastasiia Dyatlova, Ekaterina Mironova, Victoria Polyakova, Igor Kvetnoy, Inna Evsyukova, Annalucia Carbone, Ruslan Nasyrov

**Affiliations:** 1 Department of Neonatology, Saint-Petersburg State Pediatric Medical University, Litovskaya Str., 2, 194100 St. Petersburg, Russia; doivanov@yandex.ru (D.I.); vopol@yandex.ru (V.P.); rrmd99@mail.ru (R.N.); 2 Department of Medical Sciences, Division of Internal Medicine and Chronobiology Laboratory, Fondazione IRCCS Casa Sollievo della Sofferenza, 71013 San Giovanni Rotondo, Italy; g.mazzoccoli@operapadrepio.it (G.M.); annalucia.carbone@gmail.com (A.C.); 3 Department of Clinical Research, CRC Scotland & London, London E14 6JE, UK; anderson.george@rocketmail.com; 4 Department of Biogerontology, Saint Petersburg Institute of Bioregulation and Gerontology, 197110 St. Petersburg, Russia; miayy@yandex.ru (N.L.); nasya-nastasya@yandex.ru (A.D.); 5 Department of Therapy, Geriatry and Anti-Aging Medicine, Academy of Postgraduate Education, Federal Medical Biological Agency, 220013 Moscow, Russia; 6 Center of Molecular Biomedicine, Saint-Petersburg Institute of Phthisiopulmonology, Lygovsky Ave. 2-4, 191036 St. Petersburg, Russia; igor.kvetnoy@yandex.ru; 7 Department of Pathology, Saint-Petersburg State University, University Embankment, 7/9, 199034 St. Petersburg, Russia; 8 Department of Newborns’ Pathology, Ott Research Institute of Obstetrics, Gynecology and Reproductology, Mendeleyevskaya Liniya, 3, 199034 St. Petersburg, Russia; eevs@yandex.ru

**Keywords:** melatonin, embryogenesis, implantation

## Abstract

Embryogenesis is a complex multi-stage process regulated by various signaling molecules including pineal and extrapineal melatonin (MT). Extrapineal MT is found in the placenta and ovaries, where it carries out local hormonal regulation. MT is necessary for normal development of oocytes, fertilization and subsequent development of human, animal and avian embryos. This review discusses the role of MT as a regulator of preimplantation development of the embryo and its implantation into endometrial tissue, followed by histo-, morpho- and organogenesis. MT possesses pronounced antioxidant properties and helps to protect the embryo from oxidative stress by regulating the expression of the *NFE2L2, SOD1*, and *GPX1* genes. MT activates the expression of the *ErbB1, ErbB4, GJA1, POU5F1,* and *Nanog* genes which are necessary for embryo implantation and blastocyst growth. MT induces the expression of vascular endothelial growth factor (VEGF) and its type 1 receptor (VEGF-R1) in the ovaries, activating angiogenesis. Given the increased difficulties in successful fertilization and embryogenesis with age, it is of note that MT slows down ovarian aging by increasing the transcription of sirtuins. MT administration to patients suffering from infertility demonstrates an increase in the effectiveness of in vitro fertilization. Thus, MT may be viewed as a key factor in embryogenesis regulation, including having utility in the management of infertility.

## 1. Introduction

Embryogenesis of mammals is a complex, multi-stage process which includes cell division, proliferation, and differentiation, leading to embryo formation. Embryogenesis is controlled by various regulatory molecules, such as cytokines, chemokines, growth factors, and steroid hormones [1,2].

Melatonin (MT), namely N-acetyl-5-methoxytryptamine, is classically associated with the regulation of circadian rhythms via its night-time release by the pineal gland [3], following the onset of darkness [4]. Although the primary circadian clock is located in the hypothalamic suprachiasmatic nucleus (SCN), peripheral biological clocks are evident in most tissues. MT is important to the synchronization of the SCN and peripheral clocks.

MT is an antioxidant, an inducer of endogenous antioxidants (superoxide dismutase (SOD) and glutathione peroxidase [5], and an anti-inflammatory, which also optimizes mitochondria function. Its antioxidant efficacy is greater than vitamins C and E [6]. Furthermore, MT inhibits the synthesis of pro-oxidant enzymes and regulates the cell cycle [7]. High MT concentrations can be found within different mitochondria of many cell types, where it is proposed to induce sirtuins and quench mitochondrial reactive oxygen species (ROS) production [8]. Melatonin is produced in many different tissues and organs [9]. There is a growing interest in the role of extrapineal MT production across a wide range of medical conditions and early developmental processes [10,11,12,13].

Pineal MT regulates embryo and fetal development [14]. However, melatonin is produced in the mammalian embryo, within the mitochondria of the embryo [15]. Although requiring confirmation in future studies, including in human embryos, such data is challenging to the sole role of maternal, pineal MT in the regulation of fetal development. The presence of MT synthesizing enzymes is not surprising given their expression in sperm and oocytes [16,17], as well as in other early developmental tissue, including the placenta [18]. Pineal MT regulates the fetal circadian rhythm, including via the fetal SCN [14].

Circadian dysregulation in women, including as arising from night-shift work, lowers fertility and dysregulation of the menstrual cycle, as well as being thought to contribute to miscarriages [6]. It is proposed here that maternal pineal MT may act via the circadian gene, Bmal1, to disinhibit the mitochondrial pyruvate dehydrogenase complex (PDC), leading to an increase in pyruvate conversion to acetyl-CoA. Acetyl-CoA not only increases adenosine-5′-triphosphate (ATP) via mitochondrial oxidative phosphorylation, and the tricarboxylic acid (TCA) cycle, but also acts to stabilize the initial MT pathway enzyme, arylalkylamine N-acetyltransferase (AANAT), leading to an increase in embryonic MT production. Such data not only links the detrimental effects of MT production decreasing, but also the detrimental impacts of a decrease in Bmal1 on fertilization, implantation and embryogenesis [19]. Such processes are relevant in other conditions (endometriosis, immunopathology), allowing pineal melatonin to regulate mitochondrial MT production [20].

Thus, melatonin plays an important role in the maintenance of reproductive function. At the same time, the role of melatonin in embryogenesis has not been adequately studied. In this regard, the purpose of the review was to analyze the effect of melatonin on various stages of embryogenesis at the cellular and molecular levels.

## 2. Melatonin Synthesis and Signaling Ways of Embryogenesis

Aralkylamine N-acetyltransferase (AANAT) is an enzyme that is involved in the day/night rhythmic production of melatonin, by modification of serotonin. The synthesis of melatonin from serotonin occurs through two enzymatic steps. The primary chemical reaction that is catalyzed by AANAT uses two substrates, acetyl-CoA and serotonin. AANAT catalyzes the transfer of the acetyl group of Acetyl-CoA to the primary amine of serotonin, thereby producing CoA and N-acetylserotonin (NAS). In the biosynthesis of melatonin, NAS is further methylated by another enzyme, N-acetylserotonin O-methyltransferase (ASMT, HIOMT synonym) to generate melatonin. The ASMT final reaction has been suggested to be the rate-determining step in melatonin biosynthesis. Enzymes of the melatonergic pathway, such as AANAT and ASMT are present in the avian eggs, yolk and white, of Japanese quails at Hamburger–Hamilton stages 1–10, suggesting a presence and possible role of MT in the embryogenesis of this species [21].

MT regulates the earliest stages of intrauterine growth in mice [15]. Interestingly, it was shown that AANAT is mostly located in mitochondria of embryonic cells. AANAT gene knockdown prevented embryo development, which was reversed by the addition of MT. The addition MT in culture medium, in which embryonic cells with AANAT knockdown grow, reversed these disorders. MT addition reducing ROS and the number of DNA mutations are caused by oxidative stress. At the molecular level, AANAT knockdown decreased expression of Tet methylcytosine dioxygenase 2 (TET2) and DNA demethylation, while the addition of MT reversed this action, with effects mediated by TET2 transforming methylcytosine (the methylated base of DNA) into 5-hydroxymethylcytosine, which promoted embryo development [15]. It was proposed that MT preserves the mitochondrial function, providing the sufficient amount of ATP for embryo development.

The effects of pineal MT may be mediated via its induction of the circadian gene, Bmal1 [15,20]. Bmal1 has a number of effects, including suppressing pyruvate dehydrogenase kinase, leading to the disinhibition of the pyruvate dehydrogenase complex (PDC). PDC is readily incorporated into mitochondria, where it converts pyruvate to acetyl-CoA. Acetyl-CoA not only increases ATP production via the TCA cycle and oxidative phosphorylation, but is also a necessary co-substrate for AANAT and therefore, the activation of the mitochondrial melatonergic pathway, reviewed in [22]. Like MT, the loss of Bmal1 is highly detrimental to embryogenesis, including via effects in oocytes [19].

This suggests that maternal pineal melatonin may be mediating its influence on embryogenesis by upregulating the mitochondrial melatonergic pathway, via Bmal1, PDC, and acetyl-CoA. However, it should be noted that NAS is often released in various ratios with melatonin from the pineal gland, with the NAS/melatonin ratio being proposed to be relevant in a number of medical conditions, including in endometriosis [22]. NAS is also a powerful antioxidant like MT. However, it does have a number of distinct effects, including being a brain-derived neurotrophic factor (BDNF) mimic, via its capacity to activate the BDNF receptor, TrkB [23], whilst it is unknown as to whether NAS acts to regulate Bmal1. As such, variations in the maternal pineal NAS/melatonin ratio may have significant impacts on embryogenesis. A number of factors can increase the NAS/melatonin ratio, including via the backward conversion of MT to NAS by aryl hydrocarbon receptor activation by the cigarette smoke component, 2,3,7,8-Tetrachlorodibenzo-p-dioxin (TCDD), reviewed in [22].

Although clearly requiring investigation in human embryos, such processes are likely to be significant regulators of successful embryogenesis and allow for the integration of MT data with the detrimental effects of Bmal1 knockdown on embryogenesis. Such a conceptualization is also parsimonious with that proposed by Yang and colleagues, that the embryo effects of MT are mediated via an increase in mitochondrial ATP. It is also of note that pineal MT is a significant regulator of the night-time immune system, where it acts to dampen activated immune cells. The relevance of such immune cell dampening in conjunction with its embryonic effects will be interesting to determine. It is also important to note that many of MT effects can be mediated via the alpha 7 nicotinic acetylcholine receptor (α7nAChR), which is also a powerful immune regulator, modulator of embryo development [24], and is expressed on mitochondria where it acts to regulate Ca2+ influx [25]. α7 receptors are also involved in angiogenic and neurogenic activity, and have anti-apoptotic effects. As pineal MT upregulates the α7nAChR over the circadian rhythm [26], the role of the α7nAChR in modulation of the various facets of embryogenesis will be important to determine.

## 3. Melatonin’s Role in Preimplantation Embryo Development and Implantation

Embryo implantation into the endometrium remains a key process of human embryogenesis. Successful implantation depends on the molecular interactions of the endometrium and embryo from 6 to 10 days after ovulation. Both endometrium receptivity and preimplantation embryo development are important aspects on this interaction. Although endometrium receptivity has classically been seen as regulated by the ratio of steroid hormones, viz estradiol (E2) and progesterone (P4) [27], many factors can act to determine successful implantation.

The process of implantation may be seen as being comprised of 3 stages. The first stage—attachment—involves the close contact of trophectoderm cells and the epithelial cell of the endometrium. The proper orientation of the blastocyst at this point is crucial to subsequent placenta development. Integrins, especially αVβ1, and the adhesion molecule, intercellular adhesion molecule (ICAM)-1, are important regulators of trophectoderm attachment to the endometrium.

Following attachment, the trophoblast and lumenal epithelium become tightly bound in the adhesion stage. Embryo adhesion is regulated by microenvironment signals that induce the activation of adhesive proteins. Following adhesion, the blastocyst implants into the epithelium and then into the endometrial stroma, thereby connecting to the maternal vascular system. This stage is the implantation, or penetration stage, at which point, blastocysts secrete matrix metalloprotease (MMPs) enzymes for cleavage of the extracellular matrix of the maternal stroma, thereby facilitating implantation [28].

Excessive ROS negatively impacts preimplantation embryo development and implantation [29], inhibiting trophectoderm cell viability, stopping the cell cycle in phases S and G2/M, and increasing cellular apoptosis [30]. Given its high antioxidant effects, MT can be a key regulator of embryo implantation.

The MT effect on *ErbB1* and *ErbB4* genes’ expression in mouse embryonal cells was studied. ErbB1 and ErbB4 proteins serve as receptors for epidermal growth factors and take part in the regulation of gene transcription, cell proliferation, differentiation, migration, and apoptosis. The expression of *Erb* family genes is necessary for successful embryo implantation [31]. Along with the study of *ErbB1* and *ErbB4* expression, the authors investigated the MT effect on the level of intracellular ROS and antioxidant capabilities of two-cell mouse embryos. MT was shown to increase *ErbB1* and *ErbB4* expression, versus controls, which luzindol prevented. MT decreased intracellular ROS by 44–46%, versus controls, which the addition of luzindol prevented. The total antioxidant capability was also increased 1.5-fold by MT, which again, luzindol prevented. Thus, MT increased the number of trophectoderm and blastocyst cells. Overall, MT decreased ROS, and increased total antioxidant capacity, whilst stimulating blastocyst growth and elevating ErbB1 and ErbB4, both being necessary for embryo implantation [32].

The effects of MT on mitochondrial function and the regulation of sirtuins also seem of some importance. This was investigated in a study utilizing the mitochondrial toxicity of rotenone, where activated oocytes were randomly distributed into four groups: control, MT addition, rotenone addition, and rotenone plus MT. The addition of MT eliminated rotenone-induced disorders in embryo development, mitochondrial dysfunction and ROS induction, thereby reducing indicants of oxidative stress and apoptosis. MT also increased the expression of *SIRT1* and *PGC-1α* genes, both being important promoters of mitochondrial biogenesis and optimized function. The knockdown of *SIRT1,* or its pharmacological inhibition, prevented MT beneficial effects, highlighting the importance of MT-induced SIRT1 in embryo development [33].

The effect of MT on the in vitro development of microinjected pronuclear mouse embryos was also studied. MT increases blastocyst division speed and the number of its cells. When such blastocysts were implanted into mice, their pregnancy and birth rates were higher than that in the control group with pronuclear macroinjection without MT administration. Such data demonstrated that MT is an important positive regulator of successful embryogenesis. Consequently, the addition of MT can provide a new alternative approach to producing a large number of transgenic animals, an important practical application of MT in investigations involving genetic engineering [34].

Eliminating the consequences of oxidative stress is also of vital importance for embryo cryopreservation, for which MT also has utility [35]. MT was shown to increase the speed of blastocyst development in both vitrified and non-vitrified rabbit embryos at the morula stage by 17% and 12%, versus controls. The activity of glutathione-s-transferase and SOD increased with MT administration in both non-vitrified and vitrified embryos, whilst the levels of LPO and NO decreased. MT also stimulated the expression of *GJA1, NFE2L2* genes, related to embryo development, and *SOD1* gene, related to reaction to oxidative stress, in both non-vitrified and vitrified embryos. Overall, MT can promote embryo development of embryos cultivated in normal conditions as well as the development of vitrified/devitrified embryos, thereby extending the utility of cryopreservation [36].

Many MT effects are mediated via the activation of its membrane receptors, MT1 and MT2 receptors. MT1 receptor is a G protein-coupled, 7-transmembrane receptor that is responsible for melatonin effects on mammalian circadian rhythm and reproductive alterations affected by day length [37]. The receptor is an integral membrane protein that is readily detectable and localized to two specific regions of the brain. The hypothalamic suprachiasmatic nucleus appears to be involved in circadian rhythm while the hypophyseal pars tuberalis may be responsible for the reproductive effects of melatonin [38]. MT2 regulates proliferation and differentiation of osteoblasts and regulates their function in depositing bone [39]. Activation of the MT2 receptor promotes vasodilation which lowers body temperature in the extremities upon daytime administration [40]. The most notable of the functions that are largely mediated by the MT2 receptor is that of phase-shifting the internal circadian clock to entrain to the Earth’s natural light-dark cycle [41]. As noted above, the MT1 receptor has been shown to have a hand in phase-shifting but this role is secondary to that of the MT2 receptor.

The effects of varying concentrations of MT on light pollution’s influence on embryo implantation and offspring growth in mice in a model of night-time light exposure was studied. Exogenous MT increased the number of offspring by 10%. Although MT did not affect pup survival, it did increase the number of implanted embryos versus light pollution exposed controls. Additionally, MT to induces a 2-fold increase in MT1 and 4-fold increase in MT2 receptors, suggesting an increased influence of MT effects at the MT2 receptor in the regulation of reproduction [42]. Besides MT elevated p53 levels by 1.5-fold versus controls. As p53 can induce leukemia-inhibiting factor (LIF) and LIF-induced estrogen, it is a significant modulator of endometrium receptivity and subsequent implantation [43]. However, there were no differences between LIF between the two groups. It should be noted that the effects of increased p53 can be important in development, by increasing ordered proliferation and decreasing the likelihood of stem cell induction [43].

However, other authors have shown that MT application at different stages of pregnancy in mice induces LIF expression [44]. Moreover, the preventive application of MT raises the expression of two marker genes of corpus luteum in the ovaries—*StAR* and *Cyp11a1* [44]. As MT also suppresses estrogen receptor (ER)*α* activation, MT will also be acting to regulate the wider effects and levels of steroids.

Clearly, MT affords protection and regulatory influence on many aspects of fertilization. As noted above, MT is an important regulator of ovulation and oocyte development [45], protecting the ovary against oxidative stress during ovulation and slowing down ovarian aging [46,47]. Many of the MT benefits are attributed to its regulation of oxidants, oxidative stress, sirtuins induction, p53, LIF, and optimized mitochondrial function.

Exogenous MT can regulate the important points of implantation [48,49,50]. Exogenous MT increases the transcription of sirtuins (Sirt1/3/6) and prevents premature ovarian aging. Moreover, exogenous MT activates *Cat* and *Cod-1* genes’ expression and protein synthesis, which inhibit oxidative stress. Exogenous MT promotes blastocyst adhesion by upregulation of *Erb1/4* and *Erb1/3* genes. MT decreases *Cas3* and *Bax* gene expression and increases Bcl2 gene expression, which play an important role in apoptosis. MT also raises adhesion protein expression, thereby improving the speed of blastocyst implantation.

The results of randomized placebo-controlled clinical trials have demonstrated that the inclusion of MT into the assisted reproductive technologies (ART) protocol for ovarian stimulation from the first day of gonadotropin-releasing hormone administration improved the oocyte and embryo quality and pregnancy outcome in women with a low quality of oocytes and polycystic ovarian syndrome [51,52,53]. Thus, experimental studies and clinical trials confirm the protective effect of MT on oocyte quality and embryo implantation, and point to the safety of its application at the first stage of infertility treatment.

## 4. Melatonin’s Effects on Histogenesis, Morphogenesis, and Organogenesis in Different Organs and Tissues

MT is also an important regulator of development subsequent to embryogenesis, including organogenesis, fetal and neonatal development.

MT’s modulation of neural system development through calcium signaling during human embryogenesis has been indicated by the use of gene ontology and the creation of an interactive MT and MT receptor protein network [54]. This network shows MT to interact with calcium signaling, e.g., with calmodulin (CALM1), G-proteins (GNAI1, GNAI3, GNA11), protein kinase α (PRKACG, PRKACA, PRKACB, PRKAR1B, TSE1), and adenylate cyclase (ADCY2, ADCYAP1) in neural system development. Such data indicates MT to regulate calcium signals and the cAMP signaling cascade. MT also interacts with nestin, a protein marker for neural stem cells, and c-fos, which are an important role to neural gene transcription, as well as binding and inhibiting the pro-apoptotic protein, BAX, whilst increasing neuroprotective signaling via AKT1 [54]. Such effects allow MT significant transcriptional control of neuronal system development.

Human brain formation starts in the early stages of pregnancy. By the third embryonic week, fetal cells have differentiated into the ectoderm, mesoderm, and endoderm. The neural plate and then the neural tube are formed from the embryo ectoderm through cells’ transformation into neuroblasts. Subsequently, the embryo undergoes a variety of complex processes leading to the formation of the central neural system (CNS). During this period, the brain is especially susceptible to damage due to a combination of high energy consumption, low antioxidant levels, and high ROS production. MT’s antioxidant effects, induction of endogenous antioxidants and optimization of mitochondrial function helps to promote cellular homeostasis [55], in turn promoting appropriate programming of brain structures [56]. Localization of MT receptors in all the brain sections from the early stages of fetal development highlights the importance of maternal, placental and local MT to morphological maturation of the CNS [57,58].

Maternal and placental MT provides protection against DNA damage, including by the inhibition of DNA methyltransferase [59]. In a preclinical diabetes model, MT prevents the formation of neural tube defects, reduces apoptosis and stimulates the proliferation of neural progenitors by processes involving regulation of the ERK pathway [60]. The administration of MT to diabetic pregnant mice prevents lipopolysaccharide-induced neural tube defects [61]. MT’s participation in the control of epigenetic regulation of gene expression preserves the morphological and functional development program of all the embryo organs [14].

MT modulates the development of a wide array of organs and tissues, including the chick retina where it mediates its effects via MT receptors and calmodulin [62]. MT can also stimulate bone tissue development in chick embryos, as shown by the large increase in thigh bones under conditions of permanent darkness [63]. MT also suppresses the teratogenic effect of nicotine on bone development in rat embryos, [64]. Melatonin also upregulates angiogenesis including in the ovary [65], with effects mediated by an increase in VEGF and the VEGF-R1 as well as an increase in NO [66].

MT regulates and is regulated by a number of microRNAs, including some that are highly expressed in early development, including miR-451, which is proposed to contribute to alterations in MT regulation in the pathoetiology of the autism spectrum disorders [20]. As miRNAs can regulate up to 100 genes, it is likely that alterations in miRNAs will contribute to co-ordination in patterned regulation of genes co-ordinated with MT expression as well as MT effects.

Overall, such data provide clear preclinical and clinical evidence of the role of MT embryogenesis. This may be of relevance to both successful pregnancy planning and subsequent pregnancy outcomes, with the prospect of utilizing MT in the preparation of patients for ART. When administered to patients with infertility problems, MT appears to improve the outcomes of in vitro fertilization. This review provides a detailed investigation of potential mechanisms of MT’s positive effect on ovary follicles, oocytes, and granulosa cells, as well as on embryo implantation processes. The authors discuss potential application of MT in vivo by patients preparing for ART (before ovulation), which can improve the quality of oocytes. The addition of MT to culture media for oocytes in vitro will accelerate their maturation, fertilization, and the early stages of early embryo development [67].

## 5. Conclusions

**Figure 1:**
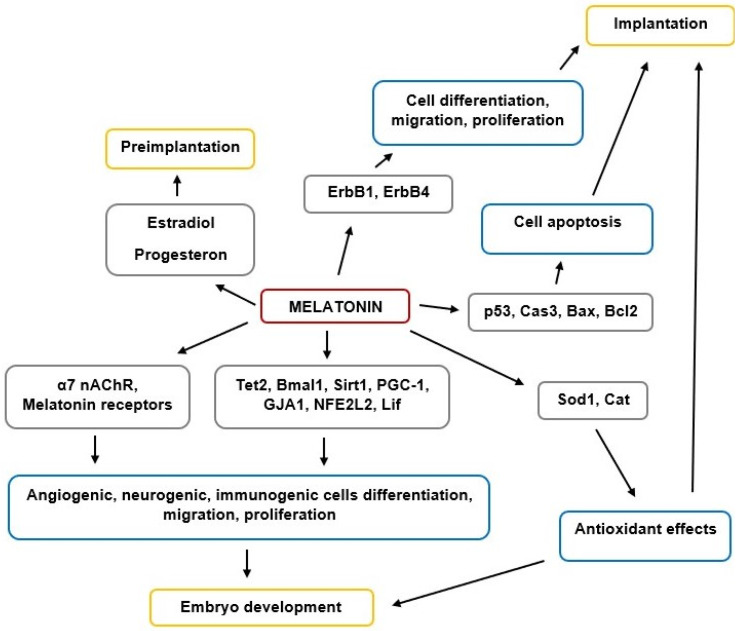
The scheme of MT’s effects on implantation and embryogenesis.

MT is a key regulator of reproductive functions in animals, including humans. The scheme of MT’s effects on implantation and embryogenesis is shown in Figure 1. MT is involved in the regulation of preimplantation by affecting the synthesis of steroid hormones—estradiol and progesterone. MT regulates the expression of the *ErbB1*, *ErbB4* genes involved in cell proliferation, migration, and differentiation at the stage of implantation. MT also regulates the expression of genes involved in apoptosis (*p53, Cas3, Bax, Bcl2*) and is important for implantation and embryo development. MT regulates the expression of genes involved in various stages of embryogenesis (*Tet2, Bmal1, Sirt1, PGC-1, GJA1, NFE2L2, Lif*). The influence of MT on embryogenesis can be mediated through the MT1, MT2, and α7NACHR receptors. An important contribution to the maintenance of implantation and embryogenesis is made by the antioxidant effect of MT, which is realized through an increase in the expression of the *Cod1* and *Cat* genes.

MT acts at many levels over the course of embryogenesis, including the preparation of the organism for pregnancy (follicle maturation, ensuring endometrium receptivity) and subsequent embryo development at various stages. MT effects include antioxidant, anti-inflammatory and mitochondria-optimizing effects, with pineal MT proposed to induce the mitochondrial melatonergic pathway, thereby optimizing mitochondrial function, sirtuin induction and endogenous antioxidant enzymes.

Endogenous MT circulating in blood binds to MT receptors on the oocyte membrane and then on the embryos. MT’s binding to MT1 and MT2 receptors activates a signaling cascade, which results in the synthesis of antioxidant enzymes and a lower level of ROS and oxidative stress, DNA demethylation, and reduced peroxide oxidation of lipids. Pineal MT may also induce mitochondria MT synthesis via Bmal1 and in co-ordination with raised levels of mitochondrial ATP production by oxidative phosphorylation and activation of the TCA cycle.

The cascade of these reactions leads to easier embryo implantation into endometrial tissue and subsequent histo- and organogenesis. Moreover, extrapineal MT produced by follicular granulosa cells promotes angiogenesis by regulating VEGF expression.

Overall, MT plays an important role in implantation and embryogenesis processes, although future research is required to clarify the importance of its various modes of action in early human development. MT is also clinically important in managing successful fertilization and pregnancy outcomes, including potential utility for infertility treatment and increasing ART effectiveness.
